# Spontaneously Generated Online Patient Experience of Modafinil: A Qualitative and NLP Analysis

**DOI:** 10.3389/fdgth.2021.598431

**Published:** 2021-02-17

**Authors:** Julia Walsh, Jonathan Cave, Frances Griffiths

**Affiliations:** ^1^Warwick Medical School, University of Warwick, Coventry, United Kingdom; ^2^Department of Economics, University of Warwick, Coventry, United Kingdom

**Keywords:** social media, natural language processing, effectiveness, causality, patient experience, evidence-based medicine, sentiment analysis, qualitative/NLP comparison

## Abstract

**Objective:** To compare the findings from a qualitative and a natural language processing (NLP) based analysis of online patient experience posts on patient experience of the effectiveness and impact of the drug Modafinil.

**Methods:** Posts (*n* = 260) from 5 online social media platforms where posts were publicly available formed the dataset/corpus. Three platforms asked posters to give a numerical rating of Modafinil. Thematic analysis: data was coded and themes generated. Data were categorized into PreModafinil, Acquisition, Dosage, and PostModafinil and compared to identify each poster's own view of whether taking Modafinil was linked to an identifiable outcome. We classified this as positive, mixed, negative, or neutral and compared this with numerical ratings. NLP: Corpus text was speech tagged and keywords and key terms extracted. We identified the following entities: drug names, condition names, symptoms, actions, and side-effects. We searched for simple relationships, collocations, and co-occurrences of entities. To identify causal text, we split the corpus into PreModafinil and PostModafinil and used n-gram analysis. To evaluate sentiment, we calculated the polarity of each post between −1 (negative) and +1 (positive). NLP results were mapped to qualitative results.

**Results:** Posters had used Modafinil for 33 different primary conditions. Eight themes were identified: the reason for taking (condition or symptom), impact of symptoms, acquisition, dosage, side effects, other interventions tried or compared to, effectiveness of Modafinil, and quality of life outcomes. Posters reported perceived effectiveness as follows: 68% positive, 12% mixed, 18% negative. Our classification was consistent with poster ratings. Of the most frequent 100 keywords/keyterms identified by term extraction 88/100 keywords and 84/100 keyterms mapped directly to the eight themes. Seven keyterms indicated negation and temporal states. Sentiment was as follows 72% positive sentiment 4% neutral 24% negative. Matching of sentiment between the qualitative and NLP methods was accurate in 64.2% of posts. If we allow for one category difference matching was accurate in 85% of posts.

**Conclusions:** User generated patient experience is a rich resource for evaluating real world effectiveness, understanding patient perspectives, and identifying research gaps. Both methods successfully identified the entities and topics contained in the posts. In contrast to current evidence, posters with a wide range of other conditions found Modafinil effective. Perceived causality and effectiveness were identified by both methods demonstrating the potential to augment existing knowledge.

## Introduction

Increasing numbers of people use social media and other online spaces as either a first or second line health information (1) and exchange resource (2, 3) with estimates suggesting the volume of online health related data will have grown by 300% between 2017 and 2020 (4). This unstructured freeform textual data contains a mass of contextually grounded detail about the perceptions and health concerns of those who post online. It has potential to add to clinical understanding, either by adding to knowledge where existing evidence is inconclusive (5), or in aiding understanding of real-world usage (6), although the methods for analyzing it are still at an early stage of development (7–13).

Although evidence based medicine (EBM) has been instrumental in raising healthcare standards and developing clinical knowledge, it has acknowledged weaknesses (14–16), including a divide between patient priorities and the research agenda (15–20) and a structural reliance on evidence from RCTs and systematic reviews (17, 18, 20, 21). Spontaneously generated online patient experience (SGOPE) is a data resource which could help address these weaknesses. However, the lack of established methodologies to analyze it inhibits its use (22–25). Natural language processing (NLP) refers to the use of computational techniques and algorithms that aim to interpret the semantic meaning from large volumes of unstructured text (26). A rapidly developing area (27), it is being used to explore health related social media usage (28–32), detecting drug or device related adverse events from user generated content (33, 34), generating new understanding about treatment switching and adherence behavior (35, 36) and as a surveillance tool for infectious disease outbreaks (37, 38) and suicide risk (12) although little work has been carried out into its use for assessing effectiveness (35).

This study was undertaken in preparation for a larger study of SGOPE data on Modafinil using NLP. Our aim was to understand the data in depth in order to develop relevant NPL analysis for the subsequent study.

Study objectives were to

- Qualitatively explore context, health conditions, and symptoms where Modafinil is used, its perceived effectiveness and impact, and identify indications of causation of effect and outcomes.
- Use NLP and corpus linguistics to identify topics, create an ontology of entities, relationships, and causal text, and evaluate overall sentiment toward perceived effectiveness of Modafinil.
- Evaluate the ability of NLP methods to identify the qualitative findings.

### Why Modafinil?

Sudden onset cognitive dysfunction and fatigue are debilitating, and distressing symptoms seen in a variety of conditions and clinical presentations. Modafinil is an out of patent oral wakefulness-promoting drug, first developed in the late 1990s, shown to be relatively safe, and with low abuse potential (39). Currently indicated only for narcolepsy in the UK (40, 41), its US FDA status enables clinicians to prescribe it “off label” to improve cognition or fatigue symptoms in many other conditions. Around 90% of its prescribed US usage is “off label” (42). Modafinil has been considered a potential therapy for a range of conditions (43), including ADHD (44), multiple sclerosis (45, 46), premature ejaculation (47), depression (48), Parkinson's disease (49), chemotherapy related fatigue (50, 51), traumatic brain injury (52), and cocaine dependence (53). Findings have been mixed, with systematic reviews generally inconclusive, showing either insufficient (52, 54–56) or low quality evidence (56–58). Previous studies have commented on the lack of research into either long term (39) or “as required” use (59). However, despite the lack of conclusive trial based evidence there appears to be a substantial amount of online discussion suggesting that there are people for whom it has made a significant difference to their symptoms and quality of life (60).

## Methods

### Study Design

**Figure 1:**
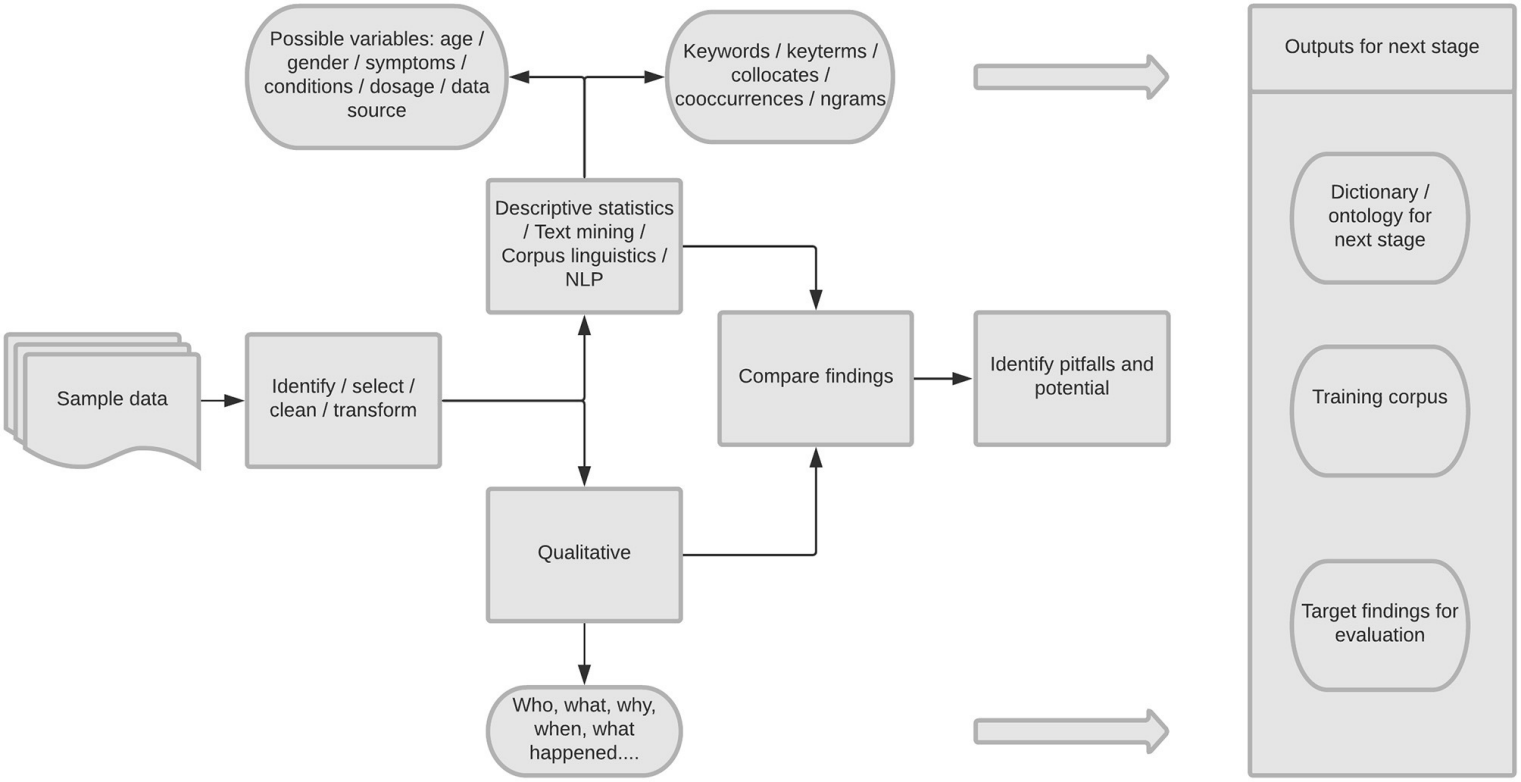
Overview of study design.

Qualitative inductive thematic analysis (61), and basic NLP analysis, of spontaneously generated online patient experience data (SGOPE) (see Figure 1). We compared the results of the NLP analysis with those from the qualitative analysis.

### Data Selection and Preparation

In January 2017, using google searches, we identified websites containing publicly available text about the experience of Modafinil use. We defined publicly available as where the data identified was available to view by anyone without any form of login, password or registration. We selected sites containing single comment “User review” posts so the type of text was similar from the different sites enabling comparison across the data sources. The final selection included: AskAPatient (62), Drugs.com (63), and WebMD (64) which provided short accounts of condition-based experiences, and Erowid (65) and ModUp where the posts were longer with greater detail of symptoms, side-effects, and self-experimentation. Online spaces can be transient and unfortunately the ModUp site no longer exists online, but all the others are still visible. From the sites we identified posts made between 1st Jan 2002 and 17th Jan 2017, and searched for individual posts about Modafinil (or variant names Provigil, Armodafinil, Nuvigil) using the site search engine. We then used random number generation to select 260 posts from across the five sites for further analysis. This volume of data was likely to be sufficient to reach data saturation for the qualitative analysis and be sufficient for linguistic analysis.

Each site had its own data structure with a variety of fields. Age and gender self-definition were optional on each of the sites. We standardized the data using the following steps:

• Standardizing field names across sources.

• Translating/encoding coded values: e.g., M/F or male/female.

• Standardizing numerical ratings scores for experience of Modafinil. Erowid and ModUp had no numerical rating; AskAPatient had a rating from 1 to 5 and drugs.com from 1 to 10 for effectiveness of Modafinil, and WebMD had ratings for effectiveness, ease of use, and satisfaction, each from 1 to 5. For the latter, the average of the three scores was calculated. We standardized all ratings to a value of between 1 and 10.

• Ages and duration of taking Modafinil, where given as an identifiable field, were grouped into ranges, and standardized across the sources.

• Posting date simplified to PostYear.

All poster identification was removed, and a unique code allocated to each post. To generate initial descriptive statistics we calculated post lengths, before coding and quantifying any included gender, age groups, duration of taking Modafinil, and numeric ratings.

### Ethical Considerations

The ethical issues surrounding the use of SGOPE data for research purposes are complex and continue to evolve (66, 67). Making a clear distinction between public and private spaces online can be difficult (68, 69). SGOPE can be classified as publicly available data (70) but as it was originally collated by the online sites and contains detail of individuals it does not fit the narrower definition of open data which can be freely used, re-used, and distributed by anyone (71). At the time of the design of this study there was a lack of clear guidance from UK Research Councils or other organizations (68, 72). In our methods we tried to minimize the potential for any form of harm.

There has been significant recent debate around expectations of privacy (73, 74). It is impossible to know the motivation, or expectation of privacy of each poster in publishing their content, but posters writing on sites that are password protected or restricted to members may have greater expectations that their privacy will be protected. Concerns exist that individuals could be identified from the posts they make, and that they may consequently suffer harm from some unforeseen use of the data. Potential harms range from unwanted commercial marketing use to profiling that could negatively impact future insurance or career choices (75). However, some studies looking at user attitudes found that social media users were generally positive toward their posts being used for research provided that they were protected from harm and that the research had potential benefit (73). There are examples of social media communities deliberately formed in open online spaces to enable individuals to come together to form a voice that is heard by health systems (76, 77).

No IP address or other geographical data was collected, all forms of usernames were removed, and the dates of the post reduced to a year value to minimize any risk of reidentification (69). Use of this type of data is covered under the doctrine of fair use (78, 79). However, we successfully arranged a data sharing agreement with AskApatient and unsuccessfully sought to put one in place with ModUp. Erowid position themselves as working with academics and medical experts and state that they generally agree to research use. However, we received no response from our repeated requests. All of the sites included invited posters to submit experience reports for publication on the respective platform. Content from drugs.com (80) and WebMD (81) carried clear messages to posters that posts were publicly viewable and could be read, collected, and used by others.

### Qualitative Analysis

Following familiarization with the posts, the data was coded and the codes merged into themes. We used MaxQDA software (82), using an iterative process of code identification and review as we progressed through the data. The coding and theme generation was done by JW, with discussion and input from FG & JC. For each theme we counted the number of posts in which they appeared.

### Evaluating Effectiveness

We categorized text within each post into one of four broad categories, PreModafinil, Acquisition, Dosage, and PostModafinil. These categories align with the base state, action, and consequence sequence required to indicate a possible perceived causal effect (83, 84) (Table 1). We compared the coded sections of each post across the sequence categories to identify the poster's own view of whether taking Modafinil was linked to an causal belief and identifiable outcome.

**Table 1:** Using categories to identify causal text and perceived effectiveness.

**Sequence**	**Post categories**	**Text describing**
Base state	PreModafinil	Symptoms + context
Action:	Acquisition/dosage	Took/did/prescribed
Consequence	PostModafinil	Effect on symptoms/side effects/context/QOL

We classified each post for perceived effectiveness (positive, mixed, negative, neutral, unclear) (Table 2). We assessed each post in isolation; balancing the positive and negative aspects of language used, reported benefits and side effects, and reference to the continued use or cessation. Fifty posts were initially independently classified by two team members and discrepancies discussed. JW then classified the remaining 210 posts.

**Table 2:** Examples of sentiment grading.

**Grade**	**Explanation**	**Example**
Positive	Overall positive. Ranged from overwhelmingly positive to indicating that benefits outweigh the disadvantages	“Significantly improved my quality-of-life with the only side effect being minor occasional headache” (2400)
Mixed	Both positive and negative effects were reported; unclear as to which sentiment prevailed	“It is a lifeline to me, but the side effects are many and do suck. Though i can honestly say, i don't find it addictive.” (1117)
Negative	Predominantly negative, usually regarding side effects.	“… feel really strange shaking overall out of sorts I am not falling asleep at work but feel so weird I'm wired that I need to find something else” (1123)
Neutral	No response or side effects noticed.	“.didn't notice any effect whatsoever, not even a side effect. “(330)

For posts which had associated numerical ratings we categorized ratings of 0–3 as negative, 4–7 as mixed, and 8–10 as positive. Using chi squared test we compared our manually assessed classification with the poster's rating.

### NLP

The narrative fields were extracted to create a corpus. Due to the small size of this exploratory dataset, we used a corpus linguistics tool, SketchEngine (85) for the structural analysis of the text. Typical NLP projects return best results from very large datasets, while corpus linguistics can be used on smaller data sets of the size also amenable to qualitative analysis. Corpus linguistics and NLP share some similar analysis techniques (86). Pre-processing for both NLP and corpus linguistics begins by dividing the text into tokens representing the smallest possible linguistic unit. Each token was assigned a part-of-speech (POS) tag from the English TreeTagger POS tagset with Sketch Engine modifications (87). We used stemming and lemmatization to assign inflected words to the same term, reducing the number of inflectional forms of a word and reducing variants to a common base (88, 89).

We used case independent word frequency and term extraction. Similar to TF-IDF of NLP, term extraction identifies the terms most specific to the text by calculating term frequency in the text compared to frequency of the same term in the reference corpus. For our reference corpus we used the English Web corpus 2013 (enTenTen13) (90), a corpus of 19 billion words collected from online sources. We extracted the top 500 specific keywords and terms. The top 100 of each indicated the most prevalent topics. The least frequent were used to identify instances of spelling variations or non-words; these were added to the domain specific dictionary intended for use in the next stage of the project.

### Entity Identification

To identify relevant entities, we used the following POS tokens tagged as nouns:

- Drug Names—both name variations of Modafinil and other drugs; those taken previously, concurrently, or subsequently in addition to some that may have no relevance to the post.
- Condition Names—identifiable condition names were categorized from term extraction analysis. Sleep related disorders were classified in line with the ICSD3 classification systems (91).
- Symptoms—symptoms of interest in this study relating to fatigue or cognitive issues. Initial dictionary entries were created from common synonyms, with further additions identified from the previous analysis.
- Action—the action of taking Modafinil has two main components: amount and frequency. Terms and phrases to identify both were found within the posts and included in the dictionary.
- Side Effects—term extraction was particularly useful in identifying side effects that the poster described, as patients often use a wide range of terms to describe them that may not map easily to recognizable medical terms.

### Relationship Identification

We used three methods to identify the relationships between entities in order to understand the semantic meaning of the text:

1. POS tagging of verbs occurring between entities to indicate simple relationships;
2. Collocation analysis (92) to reveal patterns and meanings that may not be apparent from frequency lists or manual reading of the texts;
3. Co-occurrence analysis: this assumes that if two entities co-exist within so many words that there is an underlying relationship between them. Unlike collocations, the relevant words need not be adjacent to each other, but occur within the same unit of text. Co-occurrences can highlight relationships indicating a causal link such as a side effect, outcome event, or demonstrate a negated drug event—one which denies a causal relationship between the drug and the event.

To identify possible causal text, we split the corpus into to sub corpora based on the text categories PreModafinil and PostModafinil (see section Qualitative Analysis above) and used n-gram analysis on each, looking for phrases between 3 and 5 words long that occurred at least five times in the corpora. Where an ngram was ambiguous we examined the co-location and co-occurrence analysis to assist categorization.

### Sentiment Analysis Using NLP

To evaluate sentiment we used the Python “TextBlob” package (93) to calculate the polarity of each post as a value between −1 (negative) and +1 (positive). Pre-processing included converting text to lower case, removing punctuation, and removal of the default stop words.

### Comparing the Two Methods

We manually mapped each of the 100 most frequent key words and terms from the computational corpus analysis to the themes that emerged from the qualitative analysis. Where a word/term was ambiguous or related to negation, time or scale we placed them in a separate group.

To compare NLP sentiment analysis to the qualitative categorization of positive, mixed, neutral, or negative we used two comparison scales. The first classifying a “mixed” result as being in the range ±0.01 (**Table 6**) and the second widening the “mixed” range to ±0.05 (**Table 7**). In both cases a polarity value of 0 was mapped to Neutral.

We mapped each of the 3–5 word length ngrams to the themes from the qualitative analysis. Where an ngram could apply to more than a single theme, we used the collocation and co-occurrence techniques in order to map it to the theme or group for which it was most prevalent.

We compared the NLP sentiment analysis with the qualitative analysis results for perceived effectiveness of Modafinil as follows: comparison of totals for each type of perceived effectiveness/sentiment; comparison of analysis of individual posts. The accuracy of the post level comparison was assessed using a confusion matrix.

## Results

The dataset included posts with a total length of 72,427 words (average 279; minimum 15; maximum 2,384). Posts from AskAPatient (30–417 words), Drugs.com (15–204), WebMD (29–358), Erowid (44–2,384), and ModUp (125–1,030).

Of the posters, 158/260 (61%) identified their gender and 156/260 (60%) included their age, either as an integer or as being within a range. From the two sites with 100% gender identification, there were 65% female posters on AskAPatient and 22% on Erowid. The defined age-groups ranged from under 18 to over 75, with the largest age-group being 45–54 years.

The quantifiable length of time that posters stated they had been taking Modafinil was included in 184/260 (70%) of posts. Of these 34 (18.5%) had taken it for 7 days or less, 31 (17%) 8–31 days, 61 (33.1%) for between 2 and 12 months and 58 (31.5%) for longer than 1 year.

### Qualitative Analysis

We identified eight themes which we describe below.

### Reason for Taking Modafinil

All posts were concerned with finding a solution for symptoms of fatigue, sleep and or cognitive dysfunction. Although Modafinil is only indicated for a single condition within the UK, 33 different health conditions were mentioned within this small sample of 260 posts. The most frequent were central disorders of hypersomnolence (mentioned in 26% of posts), depression (22%), sleep related breathing disorders (16%), general fatigue (9%), CFS/ME (7.5%), ADHD/ADD (6%), and MS (6%). Other conditions included cancer, traumatic brain injury, diabetes, epilepsy, fibromyalgia, autoimmune conditions, pain, IBS, hepatitis C, or post stroke fatigue. Multi-morbidity was a regular feature. While many posts referred to a single diagnosed condition, 23% referred to two concurrent conditions, 3% to three and 1.5% to four.

### Impact of Symptoms

Almost all posts contained detail of how these fatigue or cognitive symptoms affect their lives, emotionally, socially, and practically. Responses to their conditions included fear, desperation, hopelessness, resignation, embarrassment, and guilt:

*Life was miserable. I was being treated for depression and had even considered suicide. There was no way out of this rut*. [422]

*I had resigned myself to life handicapped with fatigue, and I felt really hopeless about it* [321]

Frustration was a common theme, often at their own inability to engage with “normal” life.

*I couldn't stand being this form of myself any longer—it's not me* [424]

Symptoms were described as having considerable impact on family and social relationships, putting a strain on marriages, partnerships and affecting parenting:

*My husband gets sick and tired of me being tired all the time and particularly hates it when I have to have a nap* [503]

*Before Nuvigal I couldn't keep my eyes open and live my normal life with 3 boys! Now, after Nuvigal I can actually play with my kids and be a normal mother*. [2348]

The loss, or anticipated loss of a job featured in 47 (18%) of the posts and 18 (7%) posters detailed their fear of driving, either because they had experienced falling asleep at the wheel or were concerned that they would.

### Effectiveness of Modafinil

Posts were classified as follows: 68% positive, 18% mixed, and 12% negative; four posts were neutral (see Table 2). A total of 181 posts had the potential to include a numeric rating of the effectiveness of Modafinil of which 178 posters completed the rating. The average value (after standardization) was 7.5/10. We found no significant difference between the posters numeric rating and our assessment (χ 3.3419, *p* = 0.3).

There was considerable variation in the proportion of posters reporting positive effect of Modafinil across the different sites: positive values ranged from 46 to 100%, mixed from 0 to 27%, and negative from 0 to 25% (see Table 3).

**Table 3:** Manual assessment of perceived effectiveness across data sources (% age).

	**AAP (*n* = 79)**	**DCUR (*n* = 53)**	**Erowid (*n* = 41)**	**ModUp (*n* = 38)**	**WebMd (*n* = 49)**	**All sources (*n* = 260)**
Positive	46%	72%	78%	100%	61%	67%
Mixed	27%	15%	12%	0%	27%	18%
Negative	25%	13%	7%	0%	10%	13%
No effect	1%	0%	2%	0%	0%	1%

### Impact of Effectiveness on QOL

A recurring topic among those finding Modafinil effective, was how it allowed them to return to what they felt was their personal “normal” state rather than enhancing their abilities in any way.

*This stuff is pretty amazing, i can actually have a normal day rather than fighting just to get through one. It's not what i feel but what i don't feel which is the constant fatigue, without that life has returned to “normal.”* [1388]

### Dosage

Of the 141 (55%) posts included text relating to Modafinil dosage the reported dosage taken ranged from 25 g to 1,200 mg per day in one extreme case. Although clinical guidelines usually suggest 200–400 mg daily (94), there are indications that a lower dose was found to more effective for some posters, with 17 reporting taking 100 mg/day. Tolerance was described as an issue for some, with 51 (20%) posters commenting on an apparent reduced effectiveness after weeks or months of regular daily use. Some posters reported that stopping taking Modafinil for a few days before resuming a daily dose appeared to restore its effectiveness

*After a week or so, effects not as strong and can make you feel paradoxically very tired. Take 2–3 days off, and it will resume working*. [2344]

whereas others felt it was better to take it only when they felt that they would most benefit from it:

*I did notice however that I have to take breaks from it for it to remain effective. I now only take it if I have a full day planned and have to go out, otherwise I stay at home and take a nap*. [502]

The posts also illustrated how users have experimented to find a dosage pattern that they find effective (Table 4). Almost half the posts contained text detailing the variations in frequency they had tried and those they found most effective. Comments also included the cause/effect results of experimentation of increasing or lowering the dose, taking before or after meals, with or without alcohol and how that impacted on the side effects and effectiveness

*I found if i took 50 mg every couple of days, and then 100 mg on busy days, it kept the headaches/migraines at bay*. [1117]

**Table 4:** Qualitative analysis: dosage frequency.

	**Documents**	**Percentage**	**Percentage (valid)**
Daily	53	20.38	44.92
As required	30	11.54	25.42
Twice daily	21	8.08	17.8
M-F	13	5	11.02
Unclear (variable)	10	3.85	8.47
Every other day	2	0.77	1.69
Posts with code(s)	118	45.38	100
Unspecified	142	54.62	–
Total	260	100

### Side Effects

Of the 260 posts, 128 (49%) specifically mentioned one or more side effects they considered related to the use of Modafinil. Thirty-four posts (13%) stated that they did not suffer any side effects at all, while the remaining 98 (38%) did not mention any specific side effect. Across the sample the most commonly reported side effects were headaches (57), mental health/mood related (43), appetite (30), gastric (18), urinary (16), oral (16), skin (15), cardiovascular (11), jittery (10), and difficulty sleeping (10). Other side effects including difficulty sleeping, muscular, vision effects, motor function, weight gain, tinnitus, shortness of breath, magnified pain, neuropathy, lupus flare up, swollen tongue, weight loss, and increased libido were mentioned by <10 posters. The impact of side effects varied, 12 posts described them as minimal, while 13 felt they were temporary, passing within a few days. Nine posters stated that they had stopped taking Modafinil; eight due to side effects and one because of an interaction with an MAOI antidepressant.

### Acquisition of Modafinil

Detail of how the poster found out about or acquired Modafinil was present in 136/260 (52%) posts, with 82 (31%) stating they were prescribed Modafinil by a clinician, while 54 (21%) discovered it through either their own research or via word of mouth. Difficulties in obtaining it, either within the NHS where its use is restricted to narcolepsy, or in the US where insurance companies often will not cover the cost despite clinicians prescribing it, were mentioned by 37/177 (21%) of those finding Modafinil beneficial. Self-purchasing from online sources was reported by 35 (13%) of posters:

*Now because they say Modafinil is not a bi-polar medicine they refuse to pay for it. I will not be able to afford the $650 a month. Without it I wake with nightmares. It's very sad insurance says they know better than a group of doctors and 10 years of success using a prescription* [2098]

### Other Interventions

Almost all posts included details of previously prescribed or tried interventions including self-help or lifestyle changes, and any interventions taken in combination with Modafinil. Posts often include comparative descriptors both of effect and/or side effects of the alternative intervention or combination.

*I find modafinil it more effective than caffeine although the initial effects seemed to wear off after about 8 hours or so. There are definitely less side effects than with other prescription stimulants such as phentermine or ritalin*. [2016]

### Causality

Among the 260 posts, we manually identified text relating to the perceptions of the poster's experience both pre and post Modafinil in 209 (80%). Of these, 258 (99%) contained text relating to the effect of taking Modafinil. Identification of causal text was helped by the reported rapid onset of any effect, with many posters who believe it to have an effect, either positive or negative, noticing changes within an hour of taking it.

### Comparing Qualitative and Corpus Results

Of the 100 highest frequency keywords 88 mapped directly to qualitative themes, seven related to negation or scale and 5 could not be classified. Of the 100 highest frequency key terms, 84 mapped directly to the qualitative themes, seven referred to negation and temporal aspects, and nine could not be classified (Table 5).

**Table 5:** 100 highest frequency keywords and keyterms by topic.

**Theme/type of text**	**Keyword**	**Keyterm**
Drug	Modafinil; provigil; nuvigil; armodafinil; modalert; nootropic; modafanil; modafinal; modvigil; nuvigal; modavigil; moda;	200 mg provigil;
Condition	Narcolepsy; hypersomnia; apnea; idiopathic; fatigue; fibromyalgia; cfs; insomnia;	Sleep apnea; daytime sleepiness; sleep cycle; chronic fatigue; excessive sleepiness; sleep disorder; excessive daytime sleepiness; extreme fatigue; severe sleep apnea; obstructive sleep apnea; shift work; nerve entrapment;
Symptom	Sleepiness; sleepy; tiredness; drowsiness; asleep; fatigue; drowsy; procrastination; sleep; lethargy; nap; spaciness; procrastinate; doze; irritable; tired; exhaustion	Head fog; daytime sleepiness; sleep cycle; chronic fatigue; excessive sleepiness; term memory; day time; short term memory; anxious state; afternoon fatigue; constant fatigue; brain fog;
Acquisition	Reddit; mymodafinil;	Prescription drug; sleep study;
Dosage	mg; dose; tolerance; pill;	200 mg dose; 200 mg pill; full dose; first dose; second dose; empty stomach; 100 mg dose; second pill; 200 mg provigil; 1st week; drink plenty; daily dose;
Side effect	Jittery; jitter; headache; hallucination; irritability; impulsiveness; irritable; itchiness; appetite; nausea; grouchy; clench; bpm;	Side effect; dry mouth; smelly urine; heart rate; mild anxiety; slight headache; unpleasant side; bad side; heart beat; negative side; anxious state; jittery feeling
Other drug/intervention	Stimulant; piracetam; ritalin; cpap; caffeine; amphetamine; ephedrine; adderall; adrafinil; phenylephrine; bupropion; ssri; med; pseudoephedrine; caffiene; methylphenidate; fluoxetine; cocaine;	Taking bupropion
Effect	Wakefulness; awake; alertness; euphoria; enhancer; psychoactive; nighter; schoolwork; talkative; lifesaver; palpitation; impulsiveness; amped; chatty;	Term memory; cognitive enhancer; normal sleep; productive day; mental acuity; normal sleep schedule; mental clarity; positive impact; short term memory;
Outcome		New person; normal sleep schedule; miracle drug; wonder drug;
Negation	Didn't; wasn't; hasn't; couldn't; wouldn't; hadn't	I didn't; i wasn't;
Temporal		1st week; first dose; second dose; hour period; entire day;
Scale	Hyper	
Ungrouped	Comedown; sleepless; midterm, cephalon; had	Sleep deprivation; side note; trouble sleeping; placebo effect; college student; enhancing drug; study aid; year old male; work day;

### Sentiment Analysis

The NLP TextBlob package returns sentiment polarity as a value between −1 (negative) and +1 (positive). Of the 260 posts 188 (72%) indicated positive sentiment, 10 (4%) neutral and 62 (24%) negative. The range of polarity values of posts was from −0.26 to 0.4. Tables 6, 7 show the results of comparing the classification of each method for each post. Matching was accurate in 64% of posts. If we allow for one category difference matching was accurate in 85% of posts.

**Table 6:** Sentiment analysis confusion matrix [±0.01].

	**NLP**						
**Qual**	**≥0.01**	****+ = **-0.01 to <0.01**	**0**	**≤-0.01**	**Total**		
Positive	145	2	4	23	174		Agreed evaluation
Mixed	28	3	3	13	47		1 category different
Neutral	0	0	1	3	4		2 categories different
Negative	11	4	2	18	35		Completely opposite evaluation
Total	184	9	10	57	260		
Accuracy	0.642						

*green - Agreed evaluation, beige - 1 category different, plum - 2 categories different, and red - completely opposite evaluation*.

**Table 7:** Sentiment analysis confusion matrix [±0.05].

	**NLP**						
**Qual**	**≥0.05**	****+ = **-0.05 to <0.05**	**0**	**≤-0.05**	**Total**		
Positive	130	26	4	14	174		Agreed evaluation
Mixed	24	9	3	11	47		1 category different
Neutral	0	0	1	3	4		2 categories different
Negative	9	11	2	13	35		Completely opposite evaluation
Total	163	46	10	41	260		
Accuracy	0.588						

*green - Agreed evaluation, beige - 1 category different, plum - 2 categories different, and red - completely opposite evaluation*.

The 3–5-word ngram analysis on both the pre-Modafanil (35) and post-Modafanil (106) text generated ngrams classified into the eight themes and 6 categories reported in Table 8.

**Table 8:** PreModafinil and PostModafinil 3–5-word ngrams grouped by theme.

**Theme/category**	**PreModafinil**	**PostModafinil**
Reasons	I was diagnosed with; obstructive sleep apnea; chronic fatigue syndrome; I have been; sleep apnea and; I suffer from; at the age of;	
Symptoms	I have been; that I was; I found myself; I have to; I suffer from; for the last; I was a; at the age of; and I was; I used to; I was still; I wake up; I had to	
Other interventions	I started taking;	I was on;
Acquisition	I went to; I was prescribed; I decided to	
Dosage		Early in the morning; I don't take it; I have been taking; I started taking; I take it; I don't take; I have found that; I took it; I have to; in the morning; on days that I; if I don't; when I don't; to take it
Side effects		With no side effects; I didn't notice; and I was; don't have; the next day; I don't feel; the first time I took; the side effects;
Effectiveness		I am able to; I don't feel; to be able to; get out of bed; I didn't notice; first time I took; I began to; and I was; don't have; the next day; go to sleep; I feel like I; I have found that; I was able to; I don't think; I have not; I felt like I; I used to; if I don't; that I could;
Outcome		To go to; to be able to; was able to; that I could; I felt like I;
Temporal	All the time; during the day; through the day; for the last; in the morning;	A few days; first time I took; during the day; for a few days; in the morning and; through the day; the first time
Sequential	For the last; I used to; I was still;	At the same time; as soon as I; for the first time; for a few days; the next day: I have found that; I used to; on days that I; I had to; if I don't; if I need to; the first time I took; the first time;
Negation	I don't; I didn't;	Don't have; I can not; I didn't feel; I didn't have; I didn't notice; I don't feel; I am not; I did not; I don't have; I don't know; I have not; I do not; I was not; it does not; n't be able to;
Confirmation		I was able to; I felt like I; I want to; I used to; I was on; I had to; it was a;
Ungrouped	A lot of	A lot of; hours of sleep; I need to; I have been; that I could; that I had; that I have; that I was; the rest of the; to be a; to take a
Causal ngram		I began to; I have found that; on days that I; if I don't; when I don't;

As with the keywords and keyterms we found that many of these ngrams correlated with and mapped onto the themes that emerged from the qualitative analysis. Others related specifically to temporal, sequential, negation, or confirmation text that could be used to identify phrases inferring causality. The frequently occurring ngram “*I have found that”* seen in nine posts was used to describe ways of taking the drug to maximize the effectiveness. Examples of generic ngrams and the context in which they were used are given in Table 9.

**Table 9:** Example ngrams in context.

**ngram—I have been (PreModafinil)—categorized as “Reason for taking”**
I have been battling MS Fatigue to the point of almost thinking of quitting my job, but desperately need the money.
Depression I have been working for many years with one combination after another of medications for bi polar disorder.
Before Nuvigil I have been suffering for the past 3 years or so with marked fatigue.
For the last few years I have been taking medicines to calm me down and ease my stress levels.
With that said, recently I have been back and forth to the doctor for 4 months now.
I have been fighting this for as long as my memory will take me.
I consider my own experiences to be significant in that I have been on the SSRI cipralex (celexa, escitalopram) since age 19, having experienced bouts of diagnosed major depression in my late teens.
I have been taking Provigil for about 9 months now after my sleep disorder kept me awake for 8 days even after being on a 6 mg dose of lorazepam to sleep at night for several years.
In addition to Provigil I have been on Effexor XR at 150 mg/day for my mild depression.
I have a severe lack of motivation and I have been diagnosed with ADHD.
I have been diagnosed with and suffering from Idiopathic Hypersomnia for the last 6 years.
I have been through 2 sleep studies and I wasn't quite a match for the CPAP machine but according to my doctor at the Mayo Clinic, there is obviously something wrong with how tired I am and how easily I can fall asleep.
**ngram—I used to (PreModafinil)—categorized as “Symptoms”**
I used to fight sleep all day at work, it got to the point where I was staying home because I just couldn't stay awake.
After the buzz wore off, my life became normal, which was a great improvement over the constant feelings of lethargy and helplessness I used to feel.
As someone who works online, as a writer and retailer, I used to find myself researching an article 1 min, and somehow snapping out of a haze a few hours later.
I used to drink coffee for this kind of thing, but tolerance builds up quickly and by the end of exams I'd be drinking a few cups a day and it made me feel no good.
I used to be a PhD student who was heavily dependent on Adderall for a cognitive and motivational boost.
**ngram—I have found that—(PostModafinil)—categorized as “Dosage”**
I have found that if I don't take it on the weekends that it works better.
Overall, I have found that effects of Modafinil, for me at least, are extremely subtle and almost unnoticeable until I start to think back and examine the things that I have done on a given day.
I have found that a very effective remedy is to take a couple of co-codamol tablets, which each contain 8 mg of codeine.
I have found that my own lack of worry or guilt in situations like these prevents people from becoming suspicious—nobody batted an eyelid.
The one concern I have is that I have found that cutting the dose (as I did once for several days when I didn't place the online order in time) seems to have a dramatic negative effect.
I have found that Modafinil gives me a very clear mind for problem solving.
I have found that if I skip the workout, I don't have as much energy throughout the day.
I have found that if I eat a lighter lunch, the dip is not as bad.
I have found that overall mod just works best on its own.
**ngram—the first time (PostModafinil)-categorized as “Effectiveness,” “Temporal,” and “Sequential”**
The first day on Nuvigil I felt like I had never felt before: My mind felt awake for the first time in what seems like forever.
I feel my age for the first time ever!
I have done some research and found that taking a “drug holiday” or going a day or two out of the week without it will help it stay just as potent as the first time I used it.
Like many others, the first time I took it was great!
The first time around, I nearly drove my family crazy with my talking and myself crazy trying to keep my rapid thoughts to myself.
The first time I took it, I did not have the headaches, smelly urine, post nasal drip, fuzzy vision, or muscle aches.
All I can say is that I'm now a 4.0 college student and I feel like I'm actually awake for the first time in my life.
The first time I took this (prescribed for obstructive sleep apnea) I thought “wow, this is the answer!”
At first I was skeptical that it had been the Modafinil that had caused the happiness because I tend to go through short bursts of depression and happiness and I assumed that I had just been on a good day the first time I took it but looking back I haven't had any significantly bad days while I was on Modafinil.
I am dramatically more productive at work and for the first time in my like I feel capable of planning for the future.
I will never forget how deep my mind sank that week, it was the first time I'd ever felt truly depressed—not even extended family deaths or the comedown from 220 mg of pure MDMA was as bad as how I was feeling that morning.
When I took it for an exam the first time it was amazing, I took 400mg at around 8 and stayed up the entire night studying with no problems.
I was captivated by his work, which really was excellent (we both received 1st for our efforts), but for the first time that day I was ever so slightly distracted while I was reading.
The first time, I seriously wondered about the efficacy of the things.
I think it important to note that I have never taken it every day and usually never take more than 200 mg. 600 mgs, which I took for the first time today, really has me jacked.
The first time I tried Modafinil the effect was immediate.
Right from the first time I took Modafinil not only did I feel better, but I could tell how much more I was worried about my work, the amount of detail I would put into my projects even shocked myself.
The first time I took modafinil I understood what all the hype was about.

We were able to match ngrams to the expression of causal analysis identified by the qualitative analysis (Table 10).

**Table 10:** Examples of causation reason and consequence.

**Document**	**Causation: reason**	**Causation: consequence**
1,063	***I used to*** fight sleep all day at work,	Once I started Nuvigil I have not had this problem at all, ***I feel like I*** have more energy, and my mental alertness has improved 100%.
1,090	I've tried 44 anti-depressants and only got about 25% relief	Nuvigil obliterated my depression and eased my anxiety by about 60%
1,065	Prior to provigil, I would regularly fall asleep at work or in meetings. I was afraid to drive alone for more than an hour for fear that I would fall asleep driving (I had many close calls!).	This drug has made a significant improvement in my quality of life.
1,065	I ***had felt*** (just weeks earlier) that I could not go on any further.	***I can keep*** my job and haven't wrecked any cars.
1,136	Constant feelings of lethargy and helplessness ***I used to feel***.	This drug has returned my pre-MS life to me. ***I can fully f***unction on the job
1,207	I forgot to take it 1 day	And could not stay awake and could not stop eating, just like prior to starting Provigil.

## Discussion

Within this exploratory study of the unstructured narrative post content, both methods successfully demonstrated how the majority of posters with a wide range of conditions found Modafinil effective in reducing fatigue or cognitive symptoms.

In performing the human based qualitative study first, those findings acted as an informal benchmark for the automated NLP study. The eight themes generated reflected the main aspects of patient experiences of an intervention. It also explored the detailed context that was often included within the poster's evaluation, including the reasons for starting or stopping using it, comparisons with other medications that they may have tried or moved onto, side effects and tangible or intangible effects on their quality of life.

The sample size was too small to realistically expect good results from the NLP analysis, but by using the corpus linguistics tool which used some methods found in a full NLP approach we were able to demonstrate how an NLP methodology could be used on a much larger scale to both extract topics/themes, expressions of perceived causality and evaluate effectiveness from unstructured text.

As with a recent paper comparing grounded theory with topic modeling on survey data (95), our NLP based methods successfully identified many of the qualitative findings, demonstrating how this form of data has the potential to identify effectiveness and the topics discussed within the posts. In terms of sentiment analysis, the results highlight some of the current issues with NLP methods. Although both methods show a majority of posters finding it effective for them, the confusion matrices (Tables 6, 7) highlighted some of the issues with applying generic sentiment analysis tools to health-related data. Rule based methods that determine sentiment are based on a lexicon of prelabelled words and the accuracy of the results is heavily dependent on the data that the model was trained on and the words that are considered important to that model. The majority of the existing generic NLP sentiment analysis tools were trained on either film, restaurant, or Amazon product reviews as these represent some of the largest shared annotated sentiment resources (11). Looking at some of the posts with opposing categorizations (Table 11), demonstrates how many of the concepts that posters describe in their evaluations include stopwords or words that may not be evaluated as expressing sentiment. Improved accuracy will require the development or use of a domain specific model.

**Table 11:** Example posts with conflicting sentiment analysis results.

**Manual grade negative—NLP grade positive**	**Manual grade positive—NLP grade negative**
*First day was great (started **ar** 150 dose) then falling asleep during day. Increased to 250, didn't fall asleep during day but very nervous and couldn't sleep at night. Going to breakup dosage to see if that helps Side Effects Itching, **can't** sleep at nigh*t [1146]	*I have sleep apnea and also Multiple Sclerosis. I use the 100 mg tab, but not on a daily basis. I use this when I feel tired from my MS. I recommend this for MS patients that tend to have no energy when they wake up in the mornings. It doesn't seem to affect my symptoms of MS; i.e., the tingling in my feet or legs. It just gives me energy to get **throught** the day, when I need to do what I need to do. I see my Neurologists for the medication prescription*. [5037]

### Compared to Current Evidence

These findings of overall effectiveness contrast strongly to the existing current RCT and systematic review evidence, which are generally used to determine treatment pathway options for clinicians (96). Although various RCTs have looked at Modafinil as a potential therapy across a range of conditions, findings have been mixed, and the systematic reviews generally conclude that the evidence is either inconclusive or of insufficient quality (44–47, 49, 50, 52, 53). This contradiction may have implications on both on patient care and the efficiency of healthcare provision, either through the patient not receiving an intervention that may be effective, or by receiving one that is ineffective (97, 98).

### How SGOPE Can Complement RCTs in Generating Evidence

Our results demonstrate how SGOPE can help address some of the identified issues with a research driven agenda (15) and complement RCTs. One of the possible reasons for the inconclusive trial evidence to date is the heterogeneity of effect that can occur within trials (99). Trials generally exclude participants with multiple comorbidities as these may act as confounders when measuring effectiveness (97) whereas many of the posters have two or more co-existing conditions, and may use combinations of interventions, or react to a single intervention in different ways.

Systematic reviews show how trials report either the effects of a single dose or a regular daily dose for a limited time (48, 100–102), whereas our findings include much greater variety of usage patterns. Our results illustrate how some posters have varied dosage patterns and amounts to find the optimal dosage regime for them, with some finding that lower doses than those usually prescribed were more effective. The data also demonstrated the existence of a possible tolerance effect but included the suggestion that taking occasional breaks or taking as required appeared to be a viable method of retaining effectiveness over time. Identified side effects generally reflected those already known (94), however the retrospective nature of the posts enabled the discovery of the temporary nature of some common side effects, a factor that will not be reflected in single dose trials.

### Identifying Causal Inference

Studies have begun to look at the lexical and grammatical features of causal statements in free text (84) and some work has been done using NLP to identify pharmacological adverse events from social media (33, 103, 104) suggesting that negative effectiveness can be shown from this type of data. Identifying causal text requires showing temporality; the effect occurring after the cause. Dividing the corpus into pre and post intervention by tagging the tense of tokens facilitated this classification, while ngrams and other POS tags helped us identify sequential events.

One of the issues of identifying causality in any kind of study has always been in differentiating between correlation and causation (105). Identified patterns and correlations can indicate that “something is happening” but not necessarily explain “why” (106, 107) as it does not differentiate between the causes of patterns, whether they are true, coincidental or as a result of bias. Increasing the volume and range of data may achieve a higher degree of precision and external validity (108) and while summarizing and visualization may be useful in analyzing SGOPE datasets, correlation is not the same as causation and on its own it is unlikely to be robust enough to add to an evidence base.

In our study, strength is demonstrated by how almost all posters reported an effect, either positive, negative or mixed. By using multiple data sources and including patients with a wide range of conditions we have shown consistency of findings across populations. The reported rapid onset of effect shows specificity and a biological gradient, with the cause/effect sequencing showing temporality.

The purpose of our research is not to provide a statistical proof of effectiveness across the whole patient population, but to generate a better understanding of the patient experience of using Modafinil, by exploring individual patient's perspective of whether or not it is effective for them. Causal dispositionalism is an alternative theory to the non-reductionist approach to causation, which may be relevant to this type of data. This takes a more nuanced view of how the characteristics or dispositions of both the intervention and the individual combine to affect the effectiveness (109). Rather than taking a statistically based population level view, marginal cases, and outliers are used as a starting point for further investigation of potential predicates (110). However, no matter how accurately causal text is identified, the possibility of a placebo effect, recognized as a powerful factor in a patient's assessment of effectiveness both in and out of trials (111–113) means that it is impossible to tell how much of the sentiment toward effects, either positive or negative, is due to such an effect rather than the Modafinil itself.

### Strengths and Limitations

Using content purely from the public domain is both a strength and a limitation. Although the easiest to access, it may not contain the richest patient experience data, which may be posted on sites requiring a “login.” However, using public domain data enables future replication. Validity is increased by using a diverse range of data sources. Each site comprises posts from a “community” of people who feel comfortable there, potentially leading to an element of emotional contagion between the posters (114, 115). This clustering of individuals can lead to a confirmation bias as consensus has been shown to have a positive impact on the perceived effectiveness of treatment (116). Using multiple sites can mitigate this type of contagion while the scale of the data being analyzed should negate the problems of an individual post being incorrectly classified or missed. Although there will always be an element of the unknown about the motivations and authenticity of such posts, analyzing them on a large scale rather than just a small subsection, can negate the impact of those individuals or organizations who might try to create an inaccurate impression, while techniques are continually being developed to identify spam or non-genuine posts.

As the content is generated entirely by the poster, SGOPE relies on the poster's self-description of their condition, which may include self-diagnosis, rather than that of a clinician. Reporting of symptoms and outcomes may not be as accurate or complete as it could be although this limitation can apply to any form of self-reported data, whether in a trial, clinical encounter, or online. Self-reported data, especially on hard to measure factors such as fatigue and cognition is subjective, but generally reflects the normative value of the patient. The natural, non-clinical language used within unstructured text can contain valuable information that may remain unexplored in a clinical or research setting (117), but it can also contain many spelling or grammatical errors as well as slang terms or colloquialisms that are problematic even for NLP methods created for electronic health records (EHRs) (118).

### Future Research

The next study in the project will be a fully NLP based analysis of a much larger dataset of patient experiences of Modafinil use. Having identified some of the possibilities and potential pitfalls, we will use these findings to develop methods that can be subsequently generalized to evaluate other interventions from unstructured text.

## Conclusion

We have demonstrated how SGOPE shows potential for the identification of perceived causation and evaluation of the effectiveness of Modafinil. The findings show that in comparison to the current inconclusive evidence, most posters find Modafinil to be effective in dealing with fatigue and cognitive symptoms across a wider range of conditions. Our study shows the potential for new research methods and data sources to augment existing knowledge. Although the two methods are very different, we demonstrate how computational methods can extract the same main topic areas as qualitative analysis. Although much work is needed to refine the techniques and address the challenges identified, our comparison suggests NLP can be used to look beyond the literal meaning of the words, gaining an understanding of how posters assess the effectiveness of a healthcare intervention and the outcomes that they value, on a much greater scale than is possible from qualitative studies.

## Data Availability Statement

Publicly available datasets were analyzed in this study. This data can be found at: https://github.com/jmw999/P1.

## Ethics Statement

Ethical review and approval was not required for the study on human participants in accordance with the local legislation and institutional requirements. Written informed consent for participation was not required for this study in accordance with the national legislation and the institutional requirements.

## Author Contributions

JW conceived the study design, conducted the study, and drafted the paper. FG and JC contributed to study design, advised on study conduct, and contributed to editing the paper. All authors contributed to the article and approved the submitted version.

## Conflict of Interest

The authors declare that the research was conducted in the absence of any commercial or financial relationships that could be construed as a potential conflict of interest.

## Abbreviations

EBM: Evidence based medicine
EHR: Electronic health records
NLP: Natural language processing
QOL: Quality of life
RCT: Randomized controlled trial
SGOPE: Spontaneously generated online patient experience
UGC: User generated content.
